# 3D printing injectable microbeads using a composite liposomal ink for local treatment of peritoneal diseases

**DOI:** 10.1007/s13346-023-01472-y

**Published:** 2023-11-25

**Authors:** Remo Eugster, Aymar Abel Ganguin, Amirmohammad Seidi, Simone Aleandri, Paola Luciani

**Affiliations:** Department of Chemistry, Biochemistry and Pharmaceutical Sciences, University of Bern, Freiestrasse 3, CH-3012 Bern, Switzerland

**Keywords:** Peritoneal drug delivery, Drop-on-demand manufacturing, 3D printing, Hydrogel microbeads, Sustained drug release, Liposomes

## Abstract

The peritoneal cavity offers an attractive administration route for challenging-to-treat diseases, such as peritoneal carcinomatosis, post-surgical adhesions, and peritoneal fibrosis. Achieving a uniform and prolonged drug distribution throughout the entire peritoneal space, though, is difficult due to high clearance rates, among others. To address such an unmet clinical need, alternative drug delivery approaches providing sustained drug release, reduced clearance rates, and a patient-centric strategy are required. Here, we describe the development of a 3D-printed composite platform for the sustained release of the tyrosine kinase inhibitor gefitinib (GEF), a small molecule drug with therapeutic applications for peritoneal metastasis and post-surgical adhesions. We present a robust method for the production of biodegradable liposome-loaded hydrogel microbeads that can overcome the pharmacokinetic limitations of small molecules with fast clearance rates, a current bottleneck for the intraperitoneal (IP) administration of these therapeutics. By means of an electromagnetic droplet printhead, we 3D printed microbeads employing an alginate-based ink loaded with GEF-containing multilamellar vesicles (MLVs). The sustained release of GEF from microbeads was demonstrated. In vitro studies on an immortalized human hepatic cancer cell line (Huh-7) proved concentration-dependent cell death. These findings demonstrate the potential of 3D-printed alginate microbeads containing liposomes for delivering small drug compounds into the peritoneum, overcoming previous limitations of IP drug delivery.

**Graphical abstract:**

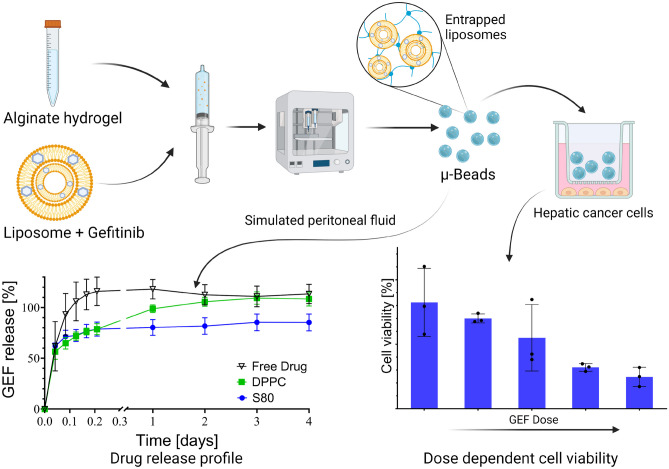

**Supplementary Information:**

The online version contains supplementary material available at 10.1007/s13346-023-01472-y.

## Introduction

The peritoneal cavity, housing key abdominal organs, is susceptible to numerous diseases including peritoneal carcinomatosis, endometriosis, and peritoneal fibrosis [1]. Current pharmacological treatments often fall short due to challenges in achieving uniform drug distribution in the peritoneal space and high clearance rates [1–3]. Small drug compounds, as well as drug carriers such as liposomes with a size below 1 µm, are cleared very efficiently from the peritoneum [4]. The common solution of increasing dosage frequency or drug dose exacerbates the risk of side effects, and the limited array of drugs and treatment schemes available for intraperitoneal (IP) use adds to the challenge [5]. Current state-of-the-art treatments, hyperthermic intraperitoneal chemotherapy (HIPEC), and early post-operative intraperitoneal chemotherapy (EPIC) require multiple re-administrations and draining over days, causing patient discomfort [5]. Hence, these challenges underscore the unmet need for new therapeutic agents and drug delivery approaches to the peritoneal cavity.

Gefitinib (GEF), a hydrophobic tyrosine kinase inhibitor, disrupts cancer cell signalling and proliferation through epidermal growth factor receptor (EGFR) binding [6], warranting its approval for the treatment of non-small cell lung cancer with specific EGFR gene mutations [7]. Recent studies have shown the treatment potential of GEF for peritoneal metastasis from various cancer types, such as ovarian and gastric cancer, as well as the potential against peritoneal adhesions that is a common complication occurring after abdominal surgery [8]. GEF has also been clinically shown to be well-tolerated barely leading to serious side effects [9], turning out to be thus a perfect drug candidate for the most common peritoneal diseases [10]. However, its hydrophobic nature and size complicate peritoneal formulation and delivery [11].

In the quest for optimizing peritoneal disease treatment, drug delivery systems should balance efficacy and convenience. Sustained drug release, a cornerstone for hydrophobic agents, can be achieved through encapsulation of therapeutic agents into liposomes, a well-established approach to obtain long-acting injectables [12]. While liposomes are effective carriers for poorly soluble compounds like GEF [11], their smaller size may not optimize retention in high-clearance environments like the peritoneal space [4]. Consequently, larger lipid-based depot systems can address this issue and enhance peritoneal retention [13]. To attain a prolonged retention time within the peritoneum (> 24 h), though, it becomes essential to exploit the benefits of larger microscale systems. Alginate hydrogels, known for their biorthogonal and biodegradable properties, offer a solution by forming microscale systems ideal for drug delivery [14–16]. Incorporating liposomes in alginate microbeads results in (1) a large surface area that enhances diffusion into the target tissue, (2) potential for more uniform drug distribution due to their ease of dispersal in the peritoneal space, and (3) a particle size that evades clearance while facilitating injectability, which underscores the suitability of liposome loaded alginate microbeads for IP drug delivery [3, 4, 17]. The hydrogel’s coherent mesh resists the release of the liposomes, prolonging their residence in the peritoneal cavity, which in turn increases drug concentration and treatment efficacy [4, 6–11, 18]. Alginate, as a biomaterial, possesses attributes of safety, biodegradability, and compatibility with 3D-printing technology and has the ability to preserve its crosslinking when exposed to the peritoneal fluid’s divalent cations [15, 19, 20]. These characteristics make it an excellent choice for creating a composite ink in the pursuit of patient-centric drug delivery systems [14, 21] While several liposome-loaded hydrogels were developed as delivery systems, they have surprisingly rarely been used in bioprinting [22] A proof-of-principle study underscores that liposomes withstand shear stress under printing conditions, bolstering advancements in this field [23]. However, besides this, the usage of liposomes in 3D printed polymers has been predominantly confined to tissue engineering or oral applications, leaving their potential for IP administration largely unexplored [24, 25].

Traditionally, hydrogel-based scaffolds containing nanocarriers are fabricated using extrusion-based methods, where nanocarriers are either adsorbed onto the surface of a 3D-printed structure or incorporated within the ink [18, 26, 27]. Incorporating the liposomes directly into the drug product ink eliminates the need for additional processing steps. Furthermore, 3D-printed drug delivery systems can be customized to achieve specific drug release profiles by varying the drug product (DP) ink design and the 3D-printed structure [27]. Research has highlighted the importance of optimizing the drug product ink and the printed scaffold design [27]. Round shapes, in particular, have been identified as favourable for drug delivery [27]. With a larger surface area than conventional gel-based systems, spherical microbeads can facilitate enhanced diffusion into the target tissue. Unlike a single large hydrogel, these microbeads can disperse more easily throughout the peritoneal space, potentially providing more uniform drug distribution [17]. Their form also facilitates injection, positioning them as an optimal choice for intraperitoneal (IP) applications [17].

The additive manufacturing of conventional 3D printing grapples with several challenges when it comes to producing spherical particles, such as achieving precise control over size, maintaining uniformity, and retaining the structural integrity of the particles [28–30]. Droplet deposition, however, has been recently proposed to overcome said challenges and form beads [31–35]. Beads might be produced by extruding a DP ink manually through a nozzle into a crosslinking solution. This mean of production, however, is largely uncontrolled being susceptible to operator influence. Also, the formation of microbeads is hardly achievable since droplets are rarely formed < 1 mm. Using a 3D printing platform for this technique, individual droplets can be expelled from a printhead only when required. This results in a finely controlled droplet-on-demand approach that facilitates a high-resolution, rapid fabrication of spherical particles [34, 35]. Electromagnetic droplet printing has been utilized to manufacture minitablets, however its potential for printing hydrogel-based microparticles containing nanocarriers for parenteral use remains largely unexplored [31].

Here, we investigate the synergy of alginate hydrogels, electromagnetic droplet printing, and liposome-based nanocarriers for peritoneal drug delivery. We aim to provide a solution that overcomes current treatment limitations, opening up new therapeutic avenues for IP drug delivery.

## Material and methods

### Materials

Gefitinib (GEF) was purchased from LC Laboratories, USA. Sodium alginate, fetal bovine serum (FBS), and L-glutamine were purchased from Sigma Aldrich, USA. Sodium acetate, acetic acid, trifluoroacetic acid (TFA), dimethyl sulfoxide (DMSO), calcium chloride, magnesium chloride, sodium chloride, 4-(2-hydroxyethyl)-1-piperazineethanesulfonic acid (HEPES), Dulbecco’s Modified Eagle Medium (DMEM), ethylenediaminetetraacetic acid (EDTA), and phosphate buffered saline (PBS) were purchased from Carl Roth, Germany. Glucose monohydrate was purchased from Hänseler, Switzerland. Chloroform was purchased from Biosolve, Netherlands. S80 (LIPOID S 80; phospholipid with 73–79% phosphatidylcholine) and DPPC (1,2-dipalmitoyl-*sn*-glycero-3-phosphocholine) were purchased from Lipoid, Germany. DiD (1,1-dioctadecyl-3,3,3,3-tetramethylindodicarbocyanine), trypsin–EDTA, and Gibco penicilin-streptomycin were purchased from Thermo Fisher, Germany. Ultrapure water with a resistivity of 18.2 MΩ⋅cm was produced by a Barnstead Smart2pure device (Thermo Fisher Scientific, Germany).

### Preparation of GEF-loaded multilamellar vesicles

Liposomal formulations were prepared by the thin film hydration method [36]. Briefly, an appropriate amount of S80 (325 mM) or DPPC (200 mM) stock solutions in chloroform were transferred in amber glass vials together with aliquots of GEF stock solutions in chloroform. For liposome preparation, GEF free base was dissolved at 100 mM. The organic solvent was evaporated under an inert nitrogen gas stream, and the lipid film was dried overnight in a desiccator. The lipid film was then rehydrated with an aqueous buffer (pH 7.1, 50 mM HEPES, 110 mM NaCl) forming multilamellar vesicles (MLVs) at a total lipid concentration of 5, 10, 20, and 30 mM. For the formation of small unilamellar vesicles (SUVs), the MLVs were subjected to 6 freeze/thaw cycles with liquid nitrogen (1 min) and a water bath at 65 °C (5 min) and extruded 10 times through a 0.2-µm polycarbonate membrane (Sterlitech, USA) using a LIPEX^®^ extruder (Evonik, Canada) at 65 °C. The particles were purified from free drug by size exclusion chromatography (SEC) using a PD MiniTrap Sephadex G-25 resin desalting column (G-25, Cytiva, USA). The purified particles were dissolved in DMSO, and the drug concentration was measured with a plate reader at an absorbance of 334 nm. Different drug-to-lipid ratios (1:5; 1:10; 1:20; 1:30) were tested keeping GEF concentration constant (1 mM). The encapsulation efficiency (EE) was calculated with the following equation:

$$\mathrm{EE}\%=\frac{{\mathrm{A}}_{334}(\mathrm{purified})}{{\mathrm{A}}_{334}(\mathrm{preG}25)}\cdot 100\mathrm{\%}$$

where “A_334_(purified)” refers to the optical density of the drug after purification with the G-25 column, and “A_334_(preG25)” refers to the optical density of the drug before free drug removal by the G-25 column. The particles’ size distribution (polydispersity index, PDI) and hydrodynamic diameter were measured using a Litesizer 500 (Anton Paar, Austria).

### Rheological characterisation of drug product ink

The rheological characteristics of the ink was investigated using a Modular Compact Rheometer MCR 72 (Anton Paar, Graz, Austria) equipped with a double gap measurement system DG26.7-SS (Anton Paar, Austria). The temperature was kept at 25 °C for all measurements. Rotatory measurements were employed to evaluate the rheological behaviour of the ink; thus, viscosity curves were obtained between a shear rate of 0.1 and 1000 s^−1^ whereas the viscosity at rest was determined by evaluating the viscosity at 0.1 s^−1^. Oscillatory measurements (amplitude sweep experiments) were used to determine the storage and loss moduli (G’ and G’’, respectively) at 1 Hz between 0.01 and 1000 Pa shear stress. The reported G’ values were obtained by averaging the first 4 G’ values as indicated in the corresponding figures (*vide infra*).

### Design of the composite drug-loaded liposomal ink and 3D printing of microbeads

Sodium alginate was dissolved in an aqueous solution (pH 7.1, 50 mM HEPES, 110 mM NaCl) overnight under stirring to reach a homogenous solution with appropriate alginate concentration. Liposomal GEF and alginate solution were mixed in a ratio of 1:1 using two syringes (B. Braun, Germany) connected with a female-female combifix adapter (B. Braun, Germany) by pushing the solution back and forth. The freshly formed ink was loaded into a cartridge (Cellink, Sweden) and was printed for crosslinking, using a Bio X Bioprinter (Cellink, Sweden) equipped with an Electro-Magnetic Droplet (EMD) printhead (Cellink, Sweden), which enables contactless jetting (drop-on-demand) into a crosslinking solution (50 mM; 135 mM CaCl_2_ + 50 mM HEPES, 110 mM NaCl) in which the microbeads were stored upon further usage. Printing parameters were set for pressure and for the opening cycle of the EMD printhead.

A multilevel full factorial design was employed to investigate the effects on the printability of the drug product ink. The factors considered were alginate concentration (1%; 2%; 3%; 4%; 5%), crosslinker concentration (50 mM; 135 mM), pressure (50 kPa; 100 kPa; 150 kPa), cycle time (20/500 ms; 1/210 ms), and printing height (10 mm; 30 mm). A total of 120 base runs were conducted to accommodate the multilevel factorial design. During each run, the specific combination of factor levels was selected according to the design matrix. The factors were manipulated independently, ensuring that their effects were isolated and evaluated accurately. The primary response variable was the printability of the spherical-shaped microbeads. For each run, the response printability was assessed by visually inspecting the printed samples and evaluating their spherical shape. The obtained data on printability (not printable = 0; spherical printlets = 1) were then subjected to statistical analysis using Minitab 18 software (Minitab, LLC, USA) with a confidence level of 95% two-sided. In order to establish the statistical significance of the relationship between the model’s terms and the response, each term’s *p*-value was evaluated against a significance level. The null hypothesis was tested through this process. Terms with a *p*-value higher than the significance level (*p* > 0.05) were deemed statistically insignificant and therefore removed from the model through stepwise regression. This methodology allowed the software to sequentially include the most impactful variable or exclude the least impactful one at every stage. Measures like S (the average distance from observed values to the regression line), R^2^, and adjusted R^2^ were utilized to assess the model’s fit to the data. The best process parameters were then determined using response optimization.

### Microbead characterization (shape fidelity evaluation)

The formed microbeads were characterized by size, shape, and morphology using light microscopy (Zeiss, Germany) and scanning electron microscopy (SEM, Gemini 450, Zeiss, Germany). Samples for light microscopy were transferred to a glass carrier plate and kept wet using a crosslinking solution to avoid them from drying. Images of the beads were analyzed using ImageJ (Fiji, 2.9.0). SEM samples were coated with gold under vacuum. The samples were then transferred onto a measurement plate and into the vacuum chamber of the system. The measurement was performed at 5 kV and magnifications ranging from × 200 to × 200,000.

### Solubility determination of GEF

To assess the kinetic solubility of GEF in peritoneal simulation fluid [19, 37, 38] (PSF: pH 7.1 (50 mM HEPES, 110 mM NaCl, 5 mM glucose, and 1.68 mM CaCl_2_) incl. 20% DMSO (v/v), GEF was dissolved in DMSO at 25 mM. Kinetic solubility was assessed by adding an excessive amount of GEF (10 µL) to PSF incl. 20% DMSO (990 µL). The mixture was further vortexed for 2 h, at 37 °C allowing the excessive GEF to precipitate. After reaching equilibrium, the samples were centrifuged (Hermle, Germany) at 12,000 g. A volume of 200 µL of supernatant was loaded in a quartz microplate (Hellma Analytics, Germany) and measured in a plate reader (Infinite MNano; Tecan, Switzerland) at an absorbance of 334 nm. While kinetic solubility refers to the maximum amount of a solid substance that can dissolve PSF + 20% DMSO under conditions where the dissolution rate is determined by the rate of mass transfer (diffusion), thermodynamic solubility determines dissolution by the equilibrium solubility. The thermodynamic solubility measurement method was derived from the United States Pharmacopoeia USP 1236 (USP 43)—solubility measurements [39]. In brief, an excess of GEF-loaded microbeads was added to PSF incl. 20% DMSO. To facilitate the dissolution of GEF, the suspension was actively mixed and incubated at 37 °C for 24 h. Equilibration time (± 5%) was verified after another 24 h. The samples were centrifuged at 12,000 g. A volume of 200 µL of supernatant was loaded in a quartz microplate and measured in a plate reader at an absorbance of 334 nm.

### Lipid entrapment and cumulative release from microbeads

Liposomes were formed as described before with the addition of 0.05 mol% DiD. To calculate the entrapment efficiency of the lipids into the microbeads, the lipid content before and after crosslinking the alginate ink was measured by fluorescence of DiD with a plate reader at an emission of 672 nm after the dye was excited at 630 nm. Therefore, the hydrogel beads were dissolved in extraction buffer (100 mM EDTA and 200 mM sodium citrate). The extracted solution was further dissolved in DMSO to destroy the liposomes, and then, DiD fluorescence was measured as described above. Cumulative lipid release was measured by horizontal incubation of DiD-liposome–loaded microbeads into a 15-mL tube containing 10 mL PSF at 37 °C and 200 rpm. At different time points, 1 mL was sampled and replaced with 1 mL PSF. The fluorescence of the collected sample was measured with a plate reader as described above. The cumulative release was normalized to the total retrieved DiD amount in the microbeads.

### Cumulative GEF release from microbeads

Microbeads containing either GEF-loaded MLVs or plain GEF were formed as described above. Cumulative GEF release was measured by horizontal incubation of the loaded microbeads into a 15-mL tube containing 10 mL PSF at 37 °C and 200 rpm. At different time points, 1 mL was sampled and replaced with 1 mL PSF. The drug concentration was measured as described. The cumulative drug release was normalized to the total amount of drug in microbeads at *t*_0_. Therefore, the drug was extracted following an established extraction protocol. In brief, extraction buffer was added to microbeads and vortexed for 20 min. To facilitate the dissolution of the microbeads the suspension was exposed to a water bath at 65 °C for 5 min. Dissolved beads were diluted (dilution factor 5) with DMSO to chloroform ratio (9:1) and once again vortexed for 15 min. After sonication for 10 min in a 65 °C water bath, samples were centrifuged at 12,000 g, and the supernatant was measured as described.

### Stability

Lipid stability and drug encapsulation were assessed over a period of 7 days. To assess the stability of the respective lipids, liposome aliquots (250 µL) were taken after purification from free drug for analysis using an HPLC equipped with a charged aerosol detector (CAD, Ultimate 3000, Thermo Fisher Scientific, USA) at *t*_0_ and *t*_7_. An HPLC method for phospholipid quantification was used [40]. In brief, liposomal (30 mM total lipid content) samples were lyophilized and resuspended in MeOH containing the internal standard (IS, palmitic acid 100 µg/mL), after undergoing a SEC at day 0 and day 7, respectively, to gain an approximate lipid concentration of 200 µg/mL. All samples were centrifuged for 10 min at 16,000 rpm. The supernatant was transferred into HPLC vials and placed in the autosampler. A Reprospher 200 column (C18, 150 × 2 mm) (Dr. Maisch GmbH, Ammerbuch-Entringen, Germany) was used at a temperature of 50 °C as stationary phase. Three different eluents, eluent A, acetonitrile + 0.2% v/v trifluoracetic acid (TFA); eluent B, methanol (MeOH) + 0.2% v/v TFA, and eluent C, ultrapure water + 0.2% v/v TFA, were used to create a linear gradient in the mobile phase. The analysis started with 25% eluent A, 65% eluent B, and 10% eluent C, at time point 0 min. After 25 min, the mobile phase was composed of 5% eluent A and 95% eluent B and kept constant for 3 min. At 28 min, the composition of eluents was brought back to the initial state during a period of 3 min, and the column was equilibrated for additional 10 min. Lipid content and chromatograms were compared to detect potential degradation. To determine GEF stability, microbeads were prepared and GEF was extracted and analyzed as described above at *t*_0_ and *t*_7_, respectively, using the same liposome stock hydrated at *t*_0_. GEF content retrieved at *t*_0_ and *t*_7_ was compared to evaluate drug degradation or diffusion into aqueous phase of the hydrated liposomes during storage at 4 °C.

### Cell culture

Huh-7 cells (RRID:CVCL_0336) were purchased from Sekisui Xenotech (Hamburg, Germany). The cells were cultured in low glucose (1.0 g/L) DMEM supplemented with 10,000 units/L of penicillin and streptomycin, 200 mM L-glutamine, and 10% (V/V) of sterile filtered (0.2 µm, cellulose acetate membrane) FBS at 37 °C in a humidified atmosphere containing 5% CO_2_. For cell splits, the cells were washed with PBS, detached with 2 mL Trypsin–EDTA (0.25%) per flask, and incubated for 4 min at 37 °C. Mycoplasma tests (MycoAlert Assay, Lonza Walkersville, Inc., USA) were performed regularly, on a 4-month basis.

### Quantitation of viable cell number in cytotoxicity assays

Huh-7 cells (passage nos. 8–12) were seeded at a density of 25,000 cells in a 48-well plate (Faust, Switzerland). Inserts for 48-well plates (CellCrown, Merck-Millipore, USA) were combined with polyamide membranes with 50-µm pores (A. Hartenstein, Germany). Cells were treated with free GEF (5, 20, 50, 100 µM), different amounts of GEF microbeads (corresponding to 5, 20, 50, and 100 µM GEF), and empty alginate microbeads for 24 h at 37 °C in a humidified atmosphere containing 5% CO_2_. To solubilize the free drug, 0.5% (V/V) of DMSO was added. The CCK-8 assay (Merck Millipore, USA) was used to determine cell viability following the manufacturer’s instructions. Briefly, the inserts were removed, the cells were washed with PBS, and 180 µL medium + 20 µL CCK-8 solution was added to each well. After a further 2 h of incubation at 37 °C and 5% CO_2_, the absorbance at 450 nm was measured with a plate reader. The cell viability was calculated with following equation:

$$\mathrm{Cell\,viability}\%= \frac{{\mathrm{OD}}_{\mathrm{treatment}}}{{\mathrm{OD}}_{\mathrm{DMEM}}} \cdot 100\%$$

where “OD treatment” refers to the optical density of the Huh-7 cells treated with the specific treatment, and “OD DMEM” refers to the optical density of Huh-7 cells treated with the vehicle (DMEM).

### Statistical analysis

All experiments were carried out in at least three replicates unless otherwise stated. The reported values are means with ± standard deviation. Microsoft Excel was used for general calculations, while GraphPad Prism 9.5 was used for plotting. Minitab 18 was used for Design of Experiments (DoE) as well as analysis and plotting of multilevel factorial design as described above.

## Results and discussion

### Microbeads preparation and physicochemical characterization 

**Fig. 1:**
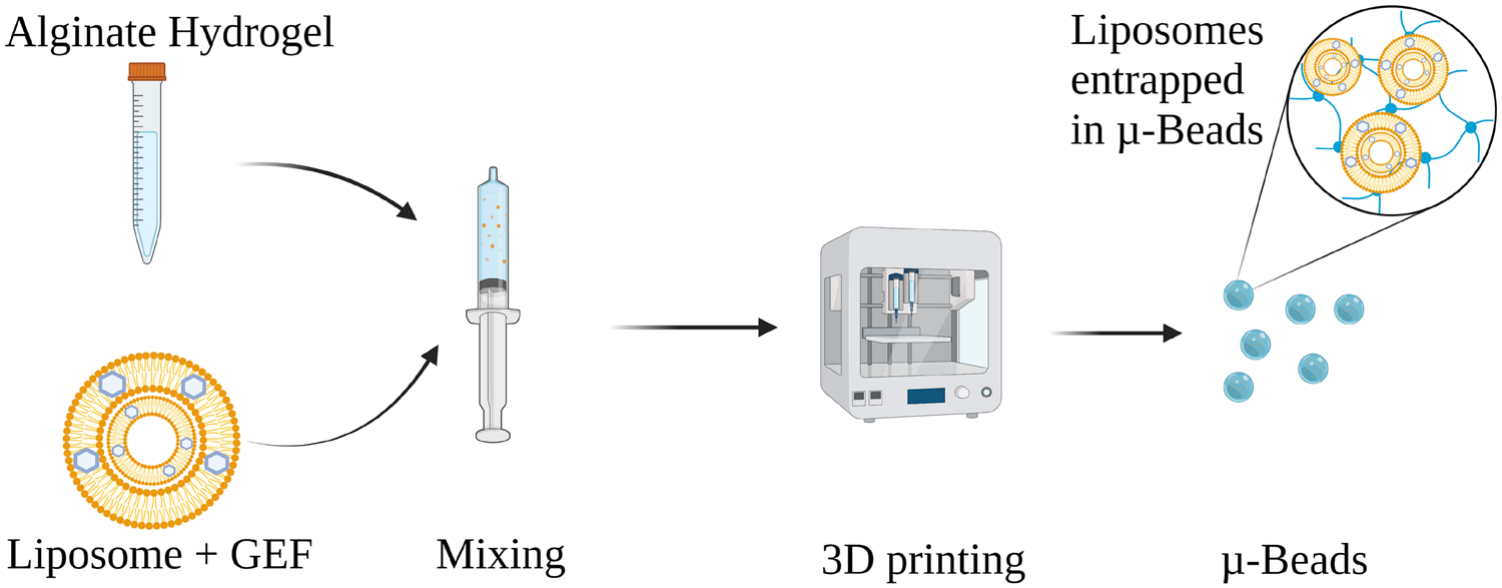
Preparation of microbeads by mixing alginate hydrogel with liposomes encapsulating GEF and 3D printing the formulation

Microbeads encapsulating GEF in two lipid formulations were prepared using drop-on-demand deposition printing for potential intraperitoneal (IP) administration, the preparation of these microbeads is presented in Fig. 1.

**Fig. 2:**
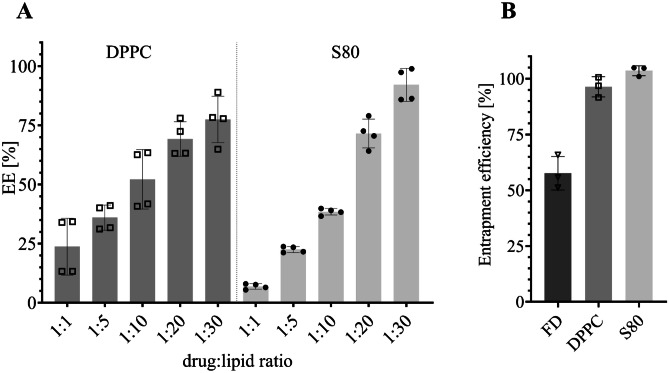
Encapsulation and entrapment efficiency. **A** Encapsulation efficiency (EE%) of GEF in DPPC or S80 MLVs with different drug-to-lipid ratios (mean ± SD, *n* = 4). **B** Entrapment efficiency of GEF-loaded beads (mean ± SD, *n* = 3)

Liposomes encapsulating the drug were prepared using the thin-film method. After hydration, excessive GEF was removed from liposomes using SEC. The main fraction containing the liposomes was used for further processing (Supporting information, Fig. S1). Liposomes were characterized in terms of their EE%. While size and PDI could have been more closely controlled by producing SUVs following F/T cycles and extrusion, we decided to formulate MLVs because of the several advantageous characteristics: due to the hydrophobic nature of the drug, low drug-to-lipid ratios yield high EE% as MLVs offer a higher hydrophobic area to accommodate the lipophilic drug within their lamellas; the large size of the MLVs (> 200 nm) is potentially beneficial considering the entrapment of the particles within the hydrogel; finally, with the hydrogel having an average mesh size ranging from 10 to 100 nm [12], particles with larger diameters should be entrapped tighter within the gel. With the aim of comparing the effect of unsaturated phospholipids on drug release, MLVs were not only formulated with DPPC but also with S80, an essential phospholipid extract from soybean containing 73–79% phosphatidylcholine. The reasons for choosing S80 were dual: its cost-effectiveness and scalability compared to DPPC being it derived from natural phospholipids [41], as well as its fibrosis-resolving features, which may be advantageous, particularly following abdominal surgery [42]. We examined various drug-to-lipid ratios for S80 and DPPC liposomes. Notably, lower drug-to-lipid ratios exhibited improved encapsulation efficiency (EE%) of the hydrophobic agent within the liposomes, as depicted in Fig. 2A. The results further revealed that S80 lipids demonstrated superior drug encapsulation efficiency (EE%) compared to other lipids, particularly at lower drug-to-lipid ratios. This suggests that the drug-to-lipid ratio played a more significant role in the encapsulation of GEF compared to the specific choice of lipids. Notably, S80 MLVs achieved a remarkable 90% encapsulation efficiency at a 1:30 ratio, surpassing the ~ 75% efficiency observed with DPPC MLVs. While using smaller drug-to-lipid ratios than 1:30 would likely result in even higher encapsulation of GEF, it is important to consider the scaling potential. To maintain GEF concentration while decreasing the drug-to-lipid ratio, substantial amounts of lipids would be required. This could pose challenges in liposome formation and lead to difficulties in the printing process due to the resulting high viscosity.

**Fig. 3:**
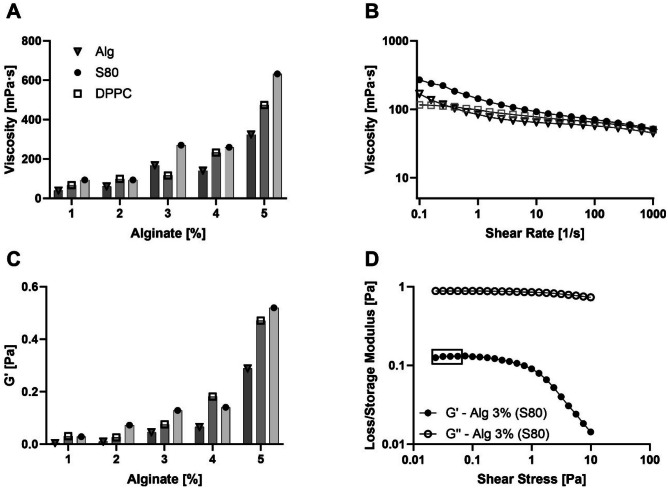
Rheological properties of liposomal ink inks. **A** Viscosity at rest for inks containing alginate spanning from 1 to 5% and either liposomes (S80 or DPPC; 15 mM) or hydration buffer. **B** Flow curve of inks used in the study (15 mM S80; 3% Alg or 15 mM DPPC; 3% Alg or 3% Alg). **C** G’ for assessed inks as for viscosity at rest. **D** Loss/storage modulus of DP ink used for in vitro study (15 mM S80; 3% Alg). Rectangle highlights values used for determining G’

The rheological characteristics of the ink, such as its viscosity at rest and its G’ values (both serve as parameters for assessing the structural strength of the material), are used as criterion to discriminate between printable and unprintable ink. All formulations exhibit shear-thinning behaviour, regardless of liposome presence (Fig. 3B and Supplementary Information Fig. S6). Moreover, the viscosity at rest increases with either rising alginate concentration or liposome addition (Fig. 3A). Inks with viscosity < 100 mPa proved excessively fluid for drop-on-demand manufacturing, resulting in continuous lines or large, uncontrollable drops instead of microbeads. Conversely, inks with viscosities > 250 mPa were too thick for extrusion at reasonable pressure, causing tear-like structures or fragmented shrapnel upon increased pressure. As for the viscosity, also G’ values (Fig. 3C) increase with rising alginate concentration and a printability window ranges from 0.046 to 0.141 Pa. All inks were viscous liquid across the measuring range, as indicated by the dominance of the viscous component (G’’) over the elastic one (G’) and the absence of a flow point (Fig. 3D).

To address the limitations of traditional 3D-printing methods for spherical structures, an EMD printhead was employed for the synthesis of microbeads. This printhead (illustrated in Supplementary information Fig. S5) utilizes an electromagnetically controlled valve to generate droplets ranging from micro- to nanolitres. The pressurized DP ink is jetted directly into a crosslinker, with the dispensing volume determined by parameters like nozzle diameter, valve speed, and actuation. Adjusting variables such as pressure, cycle time, and printhead height can influence microbead characteristics during printing. Highly viscous inks might require careful adjustment of process parameters to ensure reproducibility of the microbead printing.

A DoE strategy was used to screen either the range of formulation or process parameters that have been demonstrated to provide a printable drug product ink and a robust printing process resulting in microbeads (Supplementary information Table S1).

**Fig. 4:**
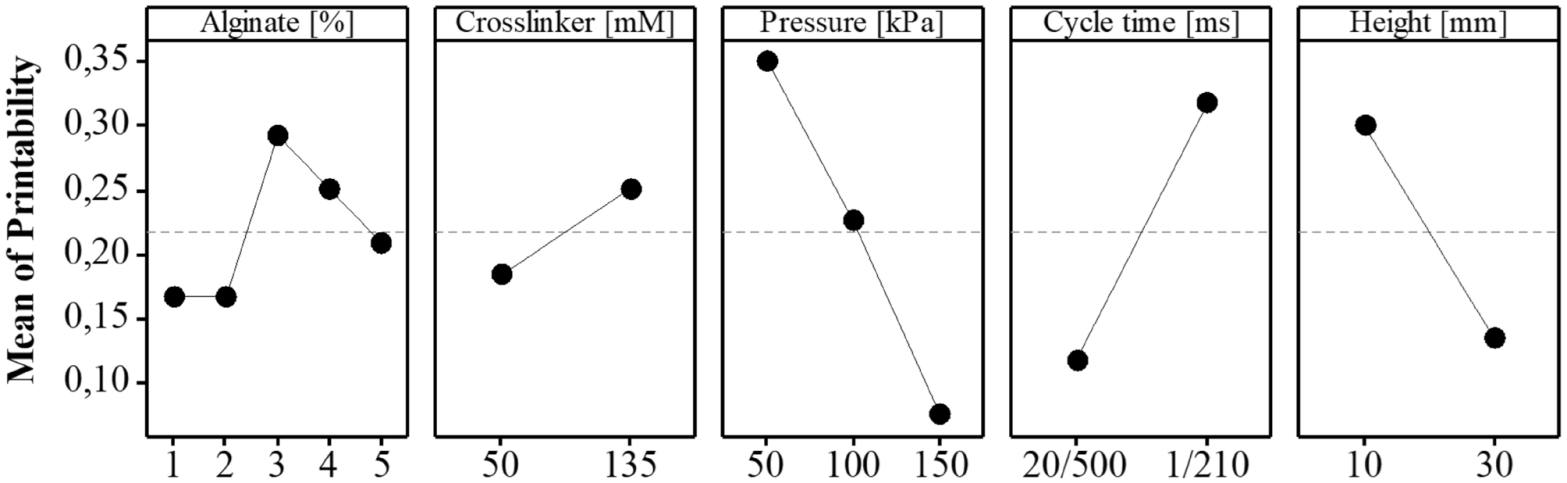
Main effects plot for printability in a multifactorial experimental design (120 runs). This plot illustrates the impact of the considered factors on the printability response. Higher response values denote a more desirable printability for each factor level. The slope of the lines connecting the factor levels serves as an indicator of the significance of their influence on the printability response

A main effects plot, as seen in Fig. 4, visually represents the average impact of independent variables, such as e.g. the alginate levels 1–5%, on the dependent variable (printability) by illustrating the factor level’s response (Supplementary information Sect. 1.1). In this context, it serves to identify how independent process parameters influence the printability of the DP ink. Thereby, the plot simplifies the relationship between independent process parameters and printability, offering a visual representation of the influence of certain process parameters on the overall printing process. The liposome-to-alginate ratio emerges as a critical factor in optimizing the printability of the DP ink. This ratio is defined as the lipid concentration divided by the alginate concentration within a specific DP ink. Throughout the study, the lipid concentration remained constant at 15 mM to ensure the delivery of the desired dose, while the alginate concentration varied from 1 to 5% (w/v), resulting in liposome-to-alginate ratios ranging from 8.8 for 1% (w/v) alginate to 39.0 for 5% (w/v). For clarity, the main effects plot displays the alginate concentrations.

After conducting the DoE with different alginate concentrations, a final concentration of 3% (w/v) was identified as the most suitable for 3D printing when combined with drug-loaded liposomes. This determination is supported by the local maximum for printability (0.29) of the alginate factor in Fig. 4. The liposome-to-alginate ratio of 13.0 (15 mM lipid; 3% alginate (w/v)), exhibited favourable printability in terms of shape retention and stability. Subsequent rheological studies revealed that a reduction in lipid content resulted in a decrease in the viscosity of the DP ink, preventing the consistent formation of droplets (Fig. 3).

For the crosslinking process, the use of CaCl_2_ was found to be most effective at 135 mM. Higher concentrations were considered favourable as they resulted in stronger crosslinking, potentially leading to a more sustained drug release profile [27]. At lower concentrations, the microbeads formed disk shapes. However, as indicated in Fig. 4, the line between the considered levels for crosslinker has a relatively low slope which indicates a smaller impact on the printability compared to other factors. In contrast, the steep slope for pressure indicates that lower pressure values are beneficial for printability. Maintaining the pressure within the range of 25–60 kPa proved to be advantageous, as excessive pressure led to the formation of tears in the printed structures. In terms of the EMD printhead, the cycle time was fine-tuned to an opening duration of less than 2 ms and a closing duration exceeding 200 ms. Extending the opening duration of the valve resulted in a hanging drop of DP ink on the printhead due to the continuous flow through the nozzle. The identified cycle time (1/200) ensured precise droplet formation and deposition during the printing process and are in accordance with the finding reported by Lu et al. [31]. Additionally, the height of the EMD print head should not exceed 10 mm from the surface of the crosslinker to avoid the formation of skewed spheres with increasing printhead height. The process parameters identified to be most suitable for drop-on-demand printing were derived from the DoE and are outlined in Table 1.

**Table 1:** Selected process parameters for drop-on-demand printing

**Alginate (% w/v)**	**Crosslinker (mM)**	**Pressure (kPa)**	**Cycle time (ms)**	**Height (mm)**
3	135	50	1/210	10

The entrapment efficiency of GEF-loaded liposomes in alginate beads is close to 100% for both, S80 and DPPC, as displayed in Fig. 2B. This indicates that only a neglectable amount of GEF was lost during the production process. As expected, the entrapment efficiency of the free drug (FD) in alginate (57.6%) is lower than in liposomal carriers (96.3% DPPC; 103.5% S80), confirming the importance of a suitable carrier to enable the delivery of the hydrophobic agent. Liposomes characterized and used on cells were always prepared freshly; nevertheless, the stability of the lipids, the drug, and its encapsulation over a period of 7 days was investigated. Regarding lipid stability for DPPC and S80, no degradation was identified over the time of observation (Supporting information, Fig. S2). Additionally, no drug degradation nor loss in GEF encapsulation was detected (Supporting information Fig. S2).

**Fig. 5:**
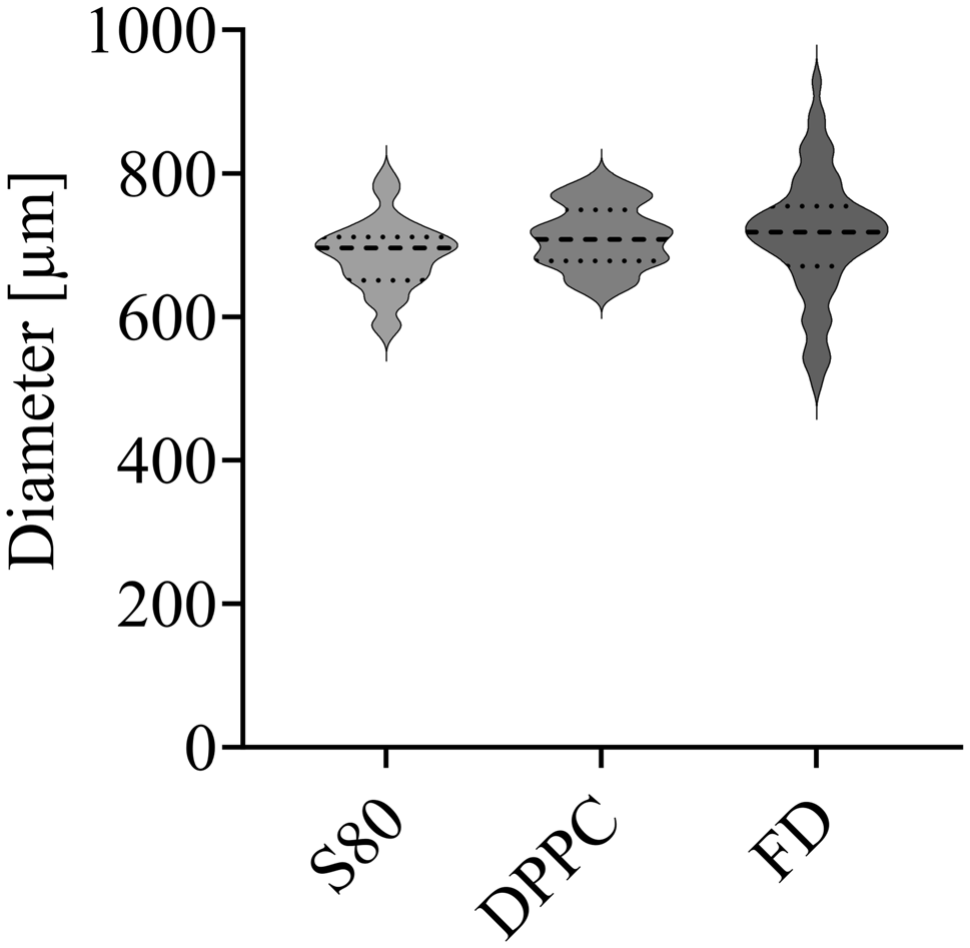
Violin plot depicting the distribution of microbead diameters across three non-consecutive batches per formulation (median ± quartile, *n* = 60). Considering intra- and inter-batch variation, this representation serves as an indicator of the robustness in the production of liposome-laden microbeads. While the liposome-laden microbeads manifest a similar distribution, the microbeads containing FD result in a larger process variation

Microbeads were characterized for their size, shape, and appearance. Microscopic measurements of the microbead’s diameter revealed the size distribution as visualized in Fig. 5 and Table 2. The distribution suggests homogeneous distribution during the production, promising aspects for future scale-up of the process. The robust process of the EMD printhead contributes significantly to the improved consistency of shape and size observed in microbeads produced through drop-on-demand manufacturing, as compared to conventional extrusion methods. Nevertheless, likely due to the lipids’ influence on the rheological properties of the ink, the presence of different lipids had an influence on the printability and, hence, on the size of the microbeads. Further, the surface-to-volume ratio (SVR) provides a measure of surface per amount of volume. In systems with a large SVR, like S80 microbeads, a higher surface area implies the potential for faster drug release. Conversely, systems with lower SVR, such as DPPC microbeads, have the ability to sustain drug release for longer periods. This distinction arises from the exposed surface area and the distance the drug must traverse through diffusion before reaching the release media.

**Table 2:** Dimensional characteristics of microbeads. Diameter and surface to volume ratio (SVR) calculated as SVR = 1/r2V (mean ± SD, *n* = 60)

**Bead**	**Diameter (µm)**	**SVR (µm**^**-1**^**)**
S80	686 ± 49	8743 ± 71
DPPC	712 ± 43	8424 ± 61
Empty	714 ± 86	8404 ± 20

**Fig. 6:**
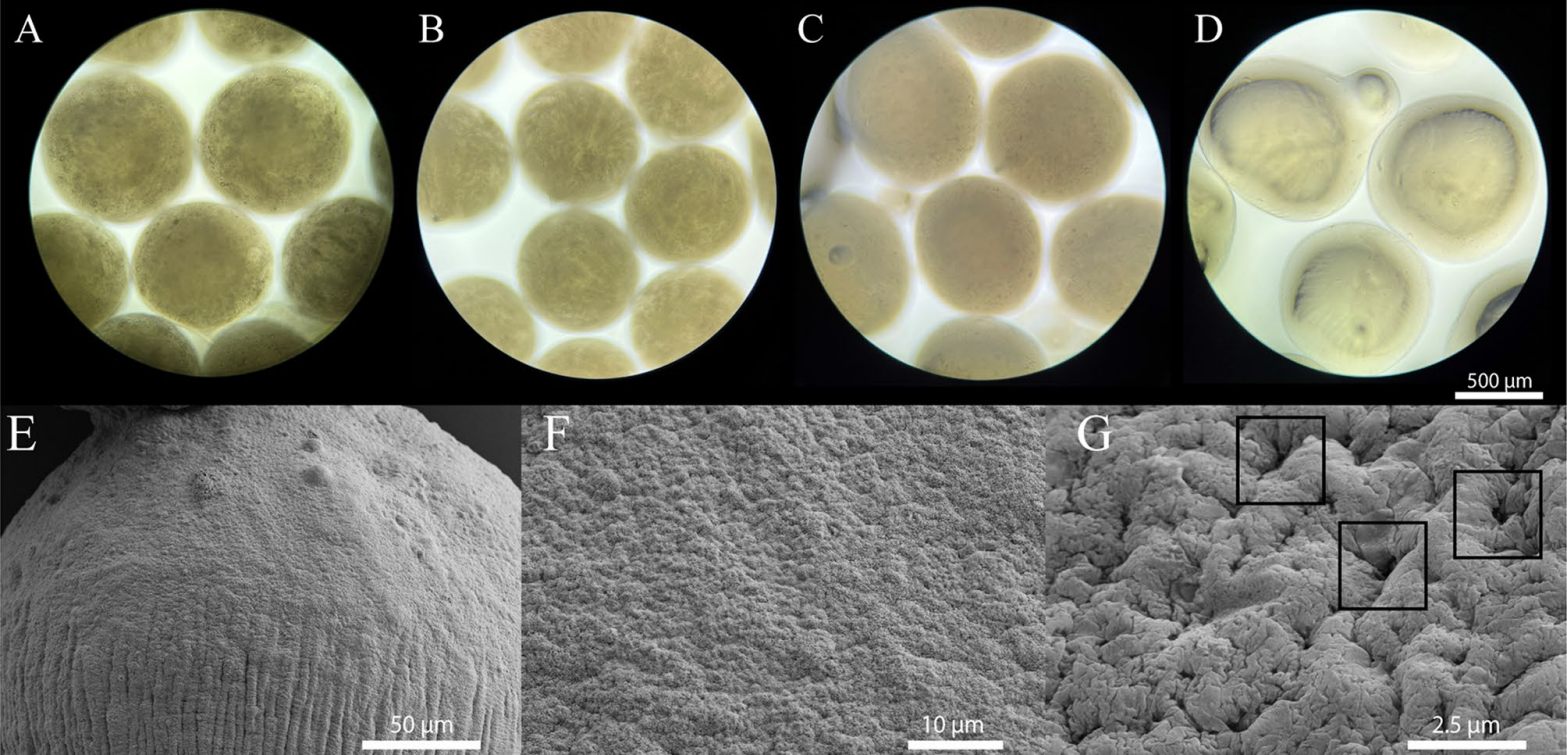
Images of microbeads after 3D printing. **A** S80 microbeads (15 mM S80; 3% Alg; 0.5 mM GEF). **B** S80 microbeads (15 mM S80; 3% Alg 0.5 mM GEF) after injection through an 18G needle. **C** DPPC microbeads (15 mM DPPC; 3% Alg; 0.5 mM GEF). **D** FD microbeads (3% Alg; 0.5 mM GEF). SEM images (**E**, **F**, **G**) of dried microbead (15 mM S80; 3% Alg; 0.5 mM GEF). **E** Surface image of microbeads at a macroscale. **F** Microscale of surface area shows homogeneous distribution of pores over surface area. **G** Pores with a diameter of ~ 100 nm on the surface indicated by rectangles

The morphological difference of the prepared formulation is observed in Fig. 6. It is apparent that S80 (A) and DPPC (C) microbeads have a spherical shape while the shape of the FD (D) microbeads has minor misshaped areas. This is most likely due to the difference in viscosity of the ink during the printing process, caused by the absence of liposomes. Also, for a minor amount of microbeads, air bubbles were entrapped into the beads as seen in Fig. 6C. All irregularities on the surface of the microbeads influence the SVR and, thereby, potentially the release of the drug since a different amount of surface is exposed to the release media. The EMD printhead facilitated the production of beads within the upper micrometres range, specifically around 700 µm in size.

Microbeads were further assessed visually for injectability through an 18G needle within which no differences or irregularities were observed indicating that the system is suitable for IP administration (Fig. 6B). Beyond the advantage of injectability, this system offers the potential for alternative administration methods. Specifically, the microbeads could be administered through a peritoneal port, a technique frequently employed in corresponding surgeries [43]. For such routes of administration, the shear stress experienced by the microbeads would be drastically reduced compared to an 18G needle (potential shear stress reduction > 60-fold). As a result, the shear stress might be estimated as it can be considered negligible. Patient-centric peritoneal delivery of large systems should be considered and, in their sum, can play a vital role in ensuring treatment success.

SEM images of the microbeads as shown in Fig. 6 provide details of the microbeads’ surface. While the beads show minor irregularities at the macroscale (E) which might be caused during printing, the surface at a microlevel seems homogeneous (F). Zooming in on the surface area, the beads’ pores were identified with a diameter of approximately 100 nm (G) which is in line with previous reporting [44]. It is assumed that said pores are small enough to allow surrounding release media to enter the beads, further increasing the exposed surface area of the bead. But at the same time, pores of 100 nm are likely capable to entrap larger MLVs and thereby hinder the lipids to be directly released for the system [45].

### Drug solubility

A solubility study of GEF in peritoneal simulation fluid (PSF) was performed to determine the most appropriate drug loading in microbeads for the subsequent drug release study in biorelevant conditions (vide infra).

Drug release from liposomes often depends on having the right environment to dissolve the drug in their surroundings [12, 46]. To gauge the solubility of GEF, we examined it in various media. For instance, we added 20% DMSO to PSF and assessed GEF solubility at 37 °C, utilizing both kinetic and thermodynamic approaches (see Table 3). PSF serves as a mimic for peritoneal fluid in individuals with peritoneal disease, covering pH, buffer capacity, glucose, MgCl_2_, and CaCl_2_ content [19]. Interestingly, we found that the best solubilities were achieved by introducing an excessive amount of GEF relative to the PSF from the delivery system, rather than relying on excessive free GEF powder dissolution. Therefore, liposomes improve the solubility of the hydrophobic agent GEF. This improved solubility can potentially lead to enhanced therapeutic effectiveness [47].

**Table 3:** Kinetic solubility of GEF vs. thermodynamic solubility of GEF obtained from microbeads (15 mM S80; 3% Alg; 0.5 mM GEF). Solubility assessments were conducted in PSF with or without 20% DMSO (mean ± SD, *n* ≥ 3)

**Media**	**Procedure**	**pH**	**GEF solubility (** $\frac{{\varvec{\mu}}\mathbf{g}}{\mathbf{m}\mathbf{L}}$**)**
PSF + 20% DMSO	Thermodynamic	7.1	23.53 ± 1.25
PSF + 20% DMSO	Thermodynamic	7.8	9.22 ± 2.03
PSF	Thermodynamic	7.1	5.20 ± 0.12
PSF + 20% DMSO	Kinetic	7.1	13.89 ± 1.07
PSF	Kinetic	7.1	0.58 ± 0.25

### Release studies

To study the microbeads’ capability to retain the loaded liposomes, hindering thus a fast peritoneal clearance, we measured the release of MLV from the system. DiD, a dye with similar lipophilic characteristics as the evaluated drug, was encapsulated in liposomes and the fluorescently labelled liposomes mixed in the ink with alginate and printed as microbeads. Given the hydrophobic nature of DiD, the fluorescence in the release medium could be considered an indicator of MLV release. Neglectable amounts (< 0.1%) of lipid release for both assessed lipids over a period of 9 days (Supporting information, Fig. S3). Similar results were obtained with microbeads filled with 200 nm SUVs, showing a neglectable liposome release for 6 days (Supporting information, Fig S4). This observation suggests that the CaCl_2_ in the release medium is capable of preserving the alginate’s integrity and, in turn, securing the liposomes, thus indicating that the surrounding environment plays a role in the liposome release process [48, 49]. This finding provides further evidence to the hypothesis that the 100-nm-sized nanopores on the surface of the microbeads are capable of retaining the MLVs within the alginate beads effectively. The negligible release of liposomes from the microbeads could thus provide the necessary hydrophobic volume to accommodate GEF in the carrier and at the same time ensure a sustained release of the drug in the peritoneal space thanks to large size (> 1 µm) of the microbeads, offering overall a clear advantage with respect to the use of plain GEF-liposomes.

**Fig. 7:**
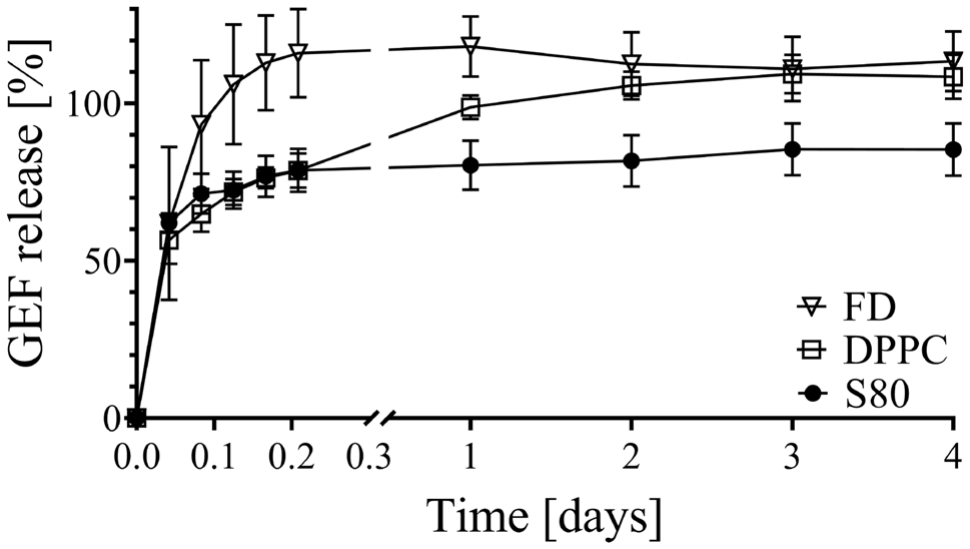
Cumulative drug release from 3D-printed microbeads (15 mM lipid; 3% Alg; 0.5 mM GEF) in PSF (mean ± SD, *n* ≥ 3). Total of 100% drug was retrieved with extraction following release experiment

Drug release from microbeads was studied in PSF with 20% DMSO at physiological temperature of 37 °C and horizontal shaking to simulate the movement within the peritoneum. Microbeads entrapping GEF-loaded DPPC and S80 liposomes were tested. As a control, FD-laden microbeads were used. Both liposomes and microbeads were stable in PSF with 20% DMSO over the assessed time as preliminary studies indicated. In absence of a liposomal carrier slowing down the diffusion of GEF, FD microbeads manifested a burst release of the drug within the first 4–5 h, as shown in (Fig. 7).

GEF release from liposomes in microbeads was sustained over 3 days reaching overall ~ 100% and ~ 80% drug release for DPPC and S80, respectively. The longer retention of GEF in the S80 system can be attributed to a potential preferential interaction between the drug and the S80 lipid bilayer, which aligns with the observed trend in encapsulation efficiency (EE%). Moreover, the sustained release is a result of the synergistic effect of both the alginate and the liposomes. As the liposomes are entrapped within the alginate mesh, the drug is gradually released from the lipidic environment into the alginate gel.

Surgical and locoregional treatment of peritoneal metastasis, particularly in cases originating from colorectal cancer, has gained widespread acceptance following the publication of favourable patient outcomes by various groups worldwide [10]. However, it is important to acknowledge that the peritoneum can be susceptible to damage resulting from factors such as surgical trauma, infection, or exposure to peritoneal dialysis fluid following these surgical interventions in the peritoneal cavity [50, 51]. In such situations, the attachment of fibroblasts to fibrin and subsequent collagen production can lead to the formation of adhesive fibrotic tissue, necessitating advanced treatment strategies [50, 51]. Even if the liposomes are entrapped in the microbeads, the lipids could potentially manifest their bioactive role once the microbeads undergo degradation, despite the prior release of the drug. Hence, the employment of S80 is notably beneficial, given its documented antifibrotic qualities as shown by our research [42]. Further, the high availability and low cost of S80 make it a desirable candidate for further drug development and scale-up efforts.

### Assessing the therapeutic potential of the microbeads in a model of human hepatic cancer

To test the suitability of our drug delivery system, we applied different concentrations of GEF to Huh-7 cells, an immortalized human hepatic carcinoma cell line, either as free drug in DMEM (0.5% V/V DMSO used as a vehicle) or encapsulated in MLVs printed as composite alginate microbeads. The Ca^2+^ (and Mg^2+^) ions present in DMEM medium were capable of stabilizing sodium alginate microbeads during the study, avoiding fast erosion of alginate and thereby burst release of the liposomes. Cells were treated for 24 h and their viability was measured after removal of the beads. To avoid unwanted toxicity due to contact between beads and cells, inserts were used to physically separate the beads from the cells, while still ensuring GEF to pass through the insert membrane and be absorbed by the Huh-7 cells. The diffusion of GEF through the insert membrane was validated experimentally.

**Fig. 8:**
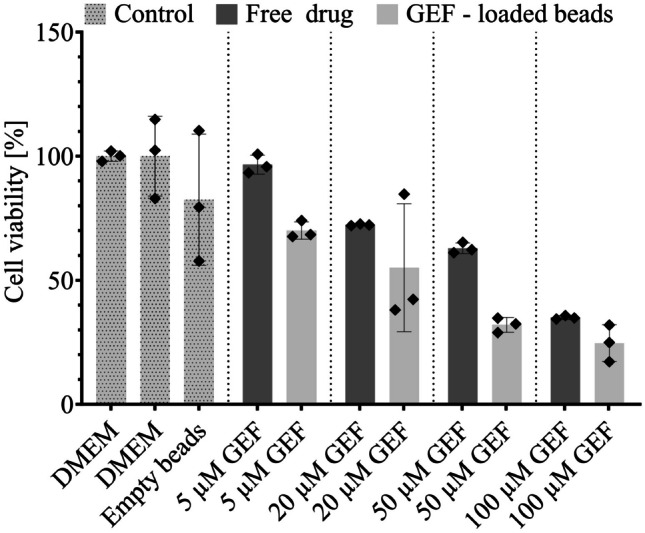
Cell viability of different GEF concentration on Huh-7 cells (mean ± SD, *n* = 3) normalized to DMEM. GEF was either administered as free drug in presence of 0.5% V/V DMSO (dark grey) or encapsulated in MLVs entrapped in microbeads (light grey), where no DMSO had to be added. Cell viability in DMEM and exposed to empty beads (light grey dotted) was measured as control. For experiments in DMEM, the solubility did not allow the testing under sink conditions

Concentration-dependent Huh-7 cell death was observed with GEF, both when in its free form and when released from the microbeads (Fig. 8). Higher cell death was obtained if the drug was released from the microbeads, which can be explained due to cytotoxicity of the beads themselves, but also due to the enhanced solubility of the drug, facilitated by the delivery system, as previously discussed. The sustained release might be enough to avoid having an excessively high free GEF concentration which would lead to precipitation, whereas albumin, the main component of FBS, probably facilitated GEF transport due to its ability of binding small hydrophobic compounds [52, 53].

Our initial, yet encouraging, in vitro results underscore the efficacy of our formulation to induce cell death without the use of organic solvents, thus suggesting a possible pathway for future in vivo studies involving GEF-loaded microbeads. The use of GEF-loaded microbeads in peritoneal chemotherapy potentially offers several advantages. Firstly, it enables localized and targeted drug delivery, minimizing systemic side effects [14, 54]. The sustained release profile of the liposome-alginate system ensures prolonged exposure of tumour cells to the drug, potentially enhancing efficacy [5]. Additionally, the use of liposomes in peritoneal cancer treatment opens possibilities for a wider range of therapeutic agents and enables to overcome limitations associated with current treatments, such as the use of charged drugs to avoid rapid clearance [5, 54].

## Conclusion

In this study, we propose a drop-on-demand manufacturing method, robust and scalable, to produce injectable microbeads using a composite liposomal ink loaded with the hydrophobic anti-cancer drug GEF. We thoroughly characterized the microbeads, revealing the presence of nanopores on their surface. We hypothesize that these nanopores enable controlled drug release from the liposomes while effectively entrapping multilamellar vesicles. Drug release experiments conducted in peritoneal simulation fluid demonstrated sustained release profiles over a 3-day period for two different lipids. The favourable release profile observed can be attributed to the interplay between alginate and the liposomes encapsulating the drug. We demonstrated a dose-dependent decrease in the viability of hepatocarcinoma cells, highlighting the potential of these microbeads as a primary treatment for cancer and metastasis within the peritoneal cavity.

Beyond chemotherapy, these delivery systems have potential applications in preventing and treating peritoneal adhesions, a common complication of abdominal surgery [3, 8, 51, 55]. By directly delivering anti-adhesion or therapeutic agents, in combination with S80, to the affected site, these systems have the ability to reduce adhesion formation and improve post-surgical outcomes as a secondary treatment [8, 42, 51, 55]. This delivery system holds potential to act as a platform for accommodating a wide range of different drugs or drug combinations, thereby potentially reducing the cost and time required for drug development. The sustained-release microbeads and drop-on-demand manufacturing could offer optimized dosing schedules and enhanced patient comfort. The delivery system may further provide an improved treatment experience compared to the patient-unfriendly regimen of repeated intraperitoneal injections or hyperthermic intraperitoneal chemotherapy (HIPEC) [5, 54]. The potential for precise customization, targeted drug release, and personalized treatment approaches suggests the transformative impact of this 3D-printed delivery system in peritoneal and controlled drug delivery.

### Supplementary Information

Below is the link to the electronic supplementary material.

Supplementary file1 (DOCX 362 KB)

## Data Availability

The authors confirm that the data supporting the findings of this study are available within the article (and/or) its supplementary materials. Raw data are available from the corresponding author (PL) on request.
