# Supplementary material for: 3D printing injectable microbeads using a composite liposomal ink for local treatment of peritoneal diseases
Source: Drug Deliv Transl Res. 2023 Nov 25;14(6):1567–81. doi: 10.1007/s13346-023-01472-y (PMC11052830; doi:10.1007/s13346-023-01472-y)
Supplement: Supplementary file 1 — Supplementary file1 (DOCX 362 KB) [file 13346_2023_1472_MOESM1_ESM.docx]

# Supporting Information

3D printing injectable microbeads using a composite liposomal ink for local treatment of peritoneal diseases

Remo Eugster, Aymar Abel Ganguin, Amirmohammad Seidi, Simone Aleandri*,* Paola Luciani^*^

*University of Bern, Department of Chemistry, Biochemistry and Pharmaceutical Sciences, Freiestrasse 3, CH-3012 Bern, Switzerland*

* To whom correspondence should be submitted: [paola.luciani@unibe.ch](mailto:Paola.luciani@unibe.ch)

## **Main effects plot**

**Fig. 4** is a main effects plot depicting the means of the dependent response variable (printability) concerning different independent variables (process parameters, such as alginate levels 1-5%). The response variable (printability) was designated values (1 and 0). Specifically, any printing attempt not resulting in spherical beads (e.g., ink running through the print head, disk-shaped or tear-shaped printlets) was considered non-printable and assigned a 0. Conversely, attempts resulting in spherical prints were assigned the value 1. This binary categorization (0 and 1) was chosen because a basis lacked to rank certain forms of failed beads over others. In our experiment, multiple independent variables (process parameters: factors with their levels, e.g., alginate 1-5%) were manipulated to observe their effects on the dependent response variable (printability).

The presented main effects plot (**Fig. 4**) serves as a graphical representation illustrating the primary effects of independent variables (process parameters: factors with their levels, e.g., alginate 1-5%) on a dependent response variable (printability). These main effects represent the average impact of each independent variable on the dependent variable, disregarding the influence of other variables. The value and magnitude/steepness of a main effect provide insights into:

The direction of the effect's steepness, indicating whether the average response value increases or decreases.

The magnitude and steepness, revealing the strength of the effect.

Consequently, the main effects plot enables visual assessment of how changes in the levels of one independent variable relate to changes in the dependent variable while keeping other variables constant.

**Table S1** Estimated model* summary for printability

| **S** | **R-sq** | **R-sq(adj)** |
| --- | --- | --- |
| 0.183 | 92.64% | 80.52% |

**F-value for the model by performing ANOVA was 7.65*


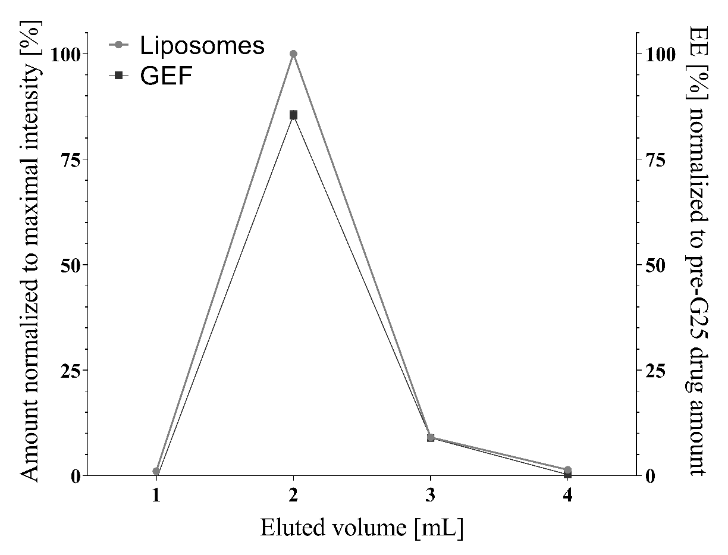


**Fig. S1** Elution profile of Liposomes (MLVs) and gefitinib from G-25 column (mean ± SD, n ≥ 3).

**
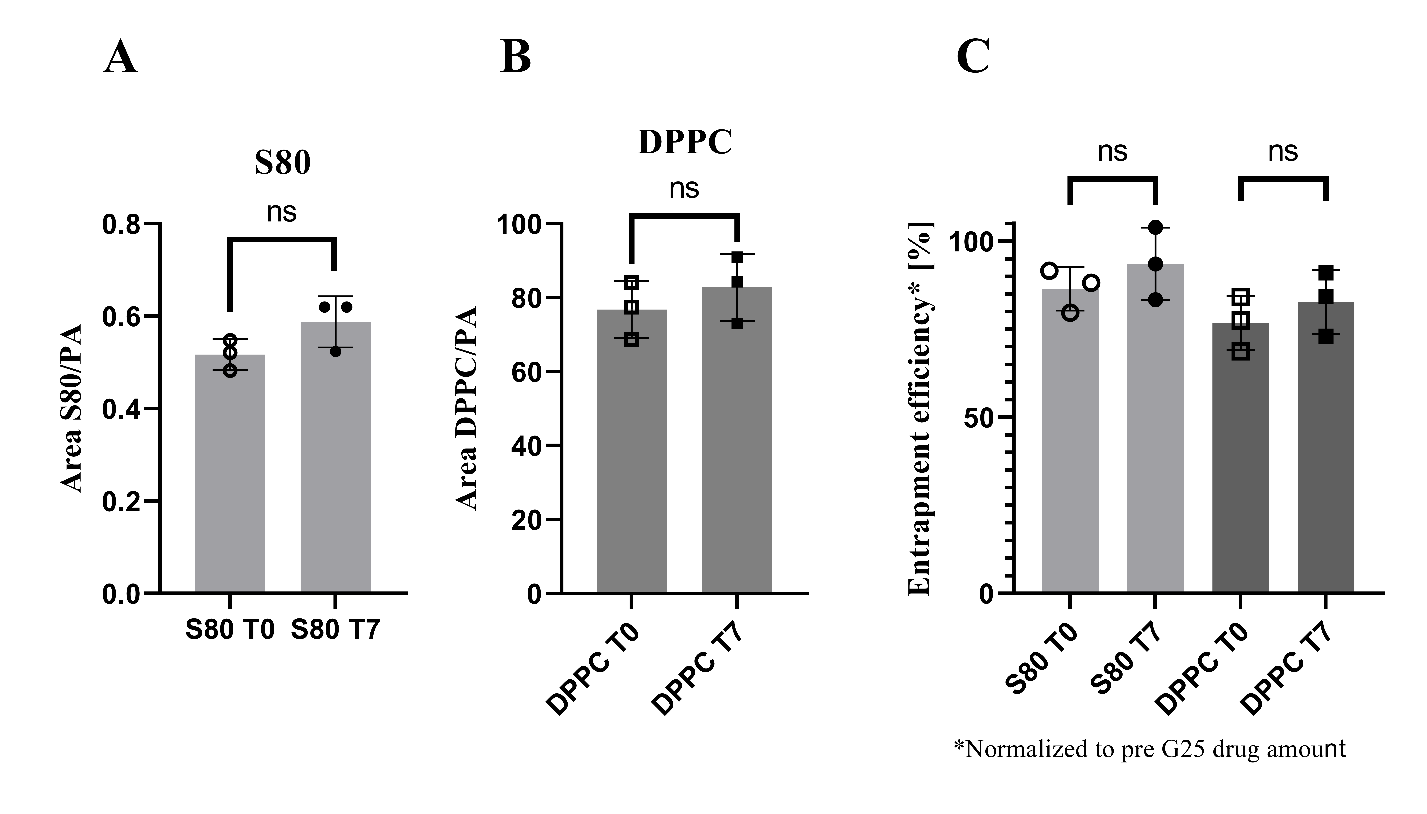
**

**Fig. S2** Stability studies of considered lipids in microbeads. A: Stability studies of S80 MLVs. The alteration in S80 content over a period of 7 days was assessed using HPLC CAD. Results are presented as the ratio of S80 to phosphoric acid (PA), where PA serves as the internal standard. B: Stability studies of DPPC MLVs. The alteration in DPPC content over a period of 7 days was assessed using HPLC CAD. Results are presented as the ratio of DPPC to PA, where PA serves as the internal standard C: Encapsulation efficiency and stability of gefitinib within MLVs over a period of 7d.


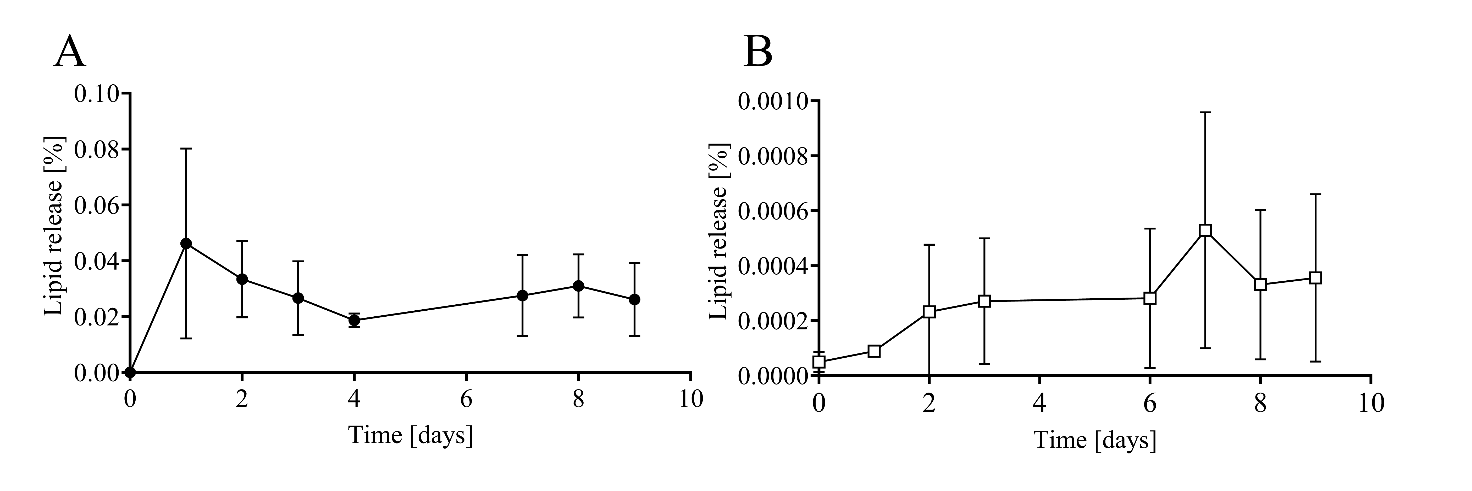


**Fig. S3** Liposomal release from microbeads considering MLVs. **A:** S80 MLV release from microbeads measured by DiD fluorescence (15 mM S80; 3% Alg; 5 nM DiD). **B:** DPPC MLV release from microbeads measured by DiD fluorescence (15 mM S80; 3% Alg; 5 nM DiD). Results normalized to cumulative content after recovery of the lipids (mean ± SD, n ≥ 3).


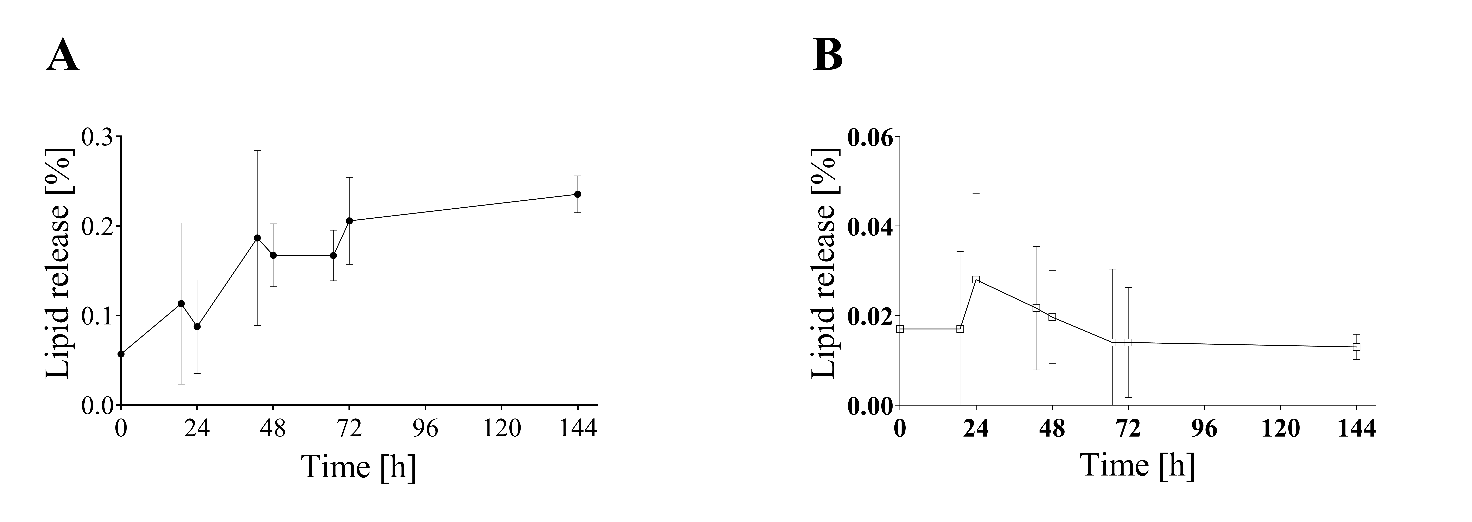


**Fig. S4** Liposomal release from microbeads considering SUVs. **A:** S80 SUV release from microbeads measured by DiD fluorescence (15 mM S80; 3% Alg; 5 nM DiD). **B:** DPPC SUV release from microbeads measured by DiD fluorescence (15 mM S80; 3% Alg; 5 nM DiD). Results normalized to cumulative content after recovery of the lipids (mean ± SD, n ≥ 3).


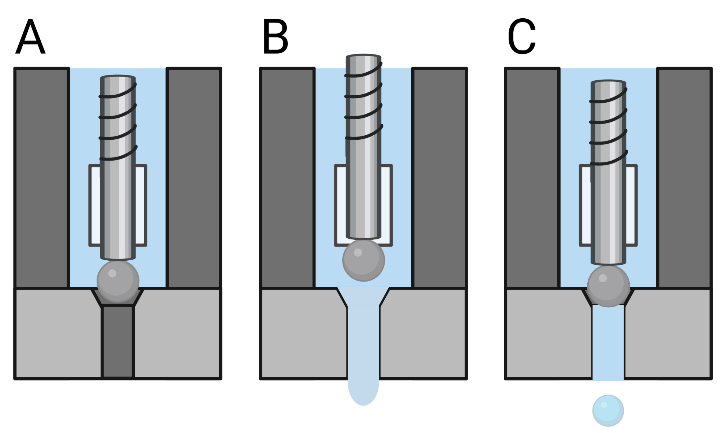


**Fig. S5**  Stages of Drop-on-Demand Printing cycle using an EMD printhead. A: The closed valve obstructs the pressurized drug product ink from reaching the nozzle. B: The opening of the valve enables the passage of drug product ink through the nozzle. C: The subsequent closing of the valve triggers droplet formation. (Adapted from the Bio X EMD Printheads User Manual by Cellink)


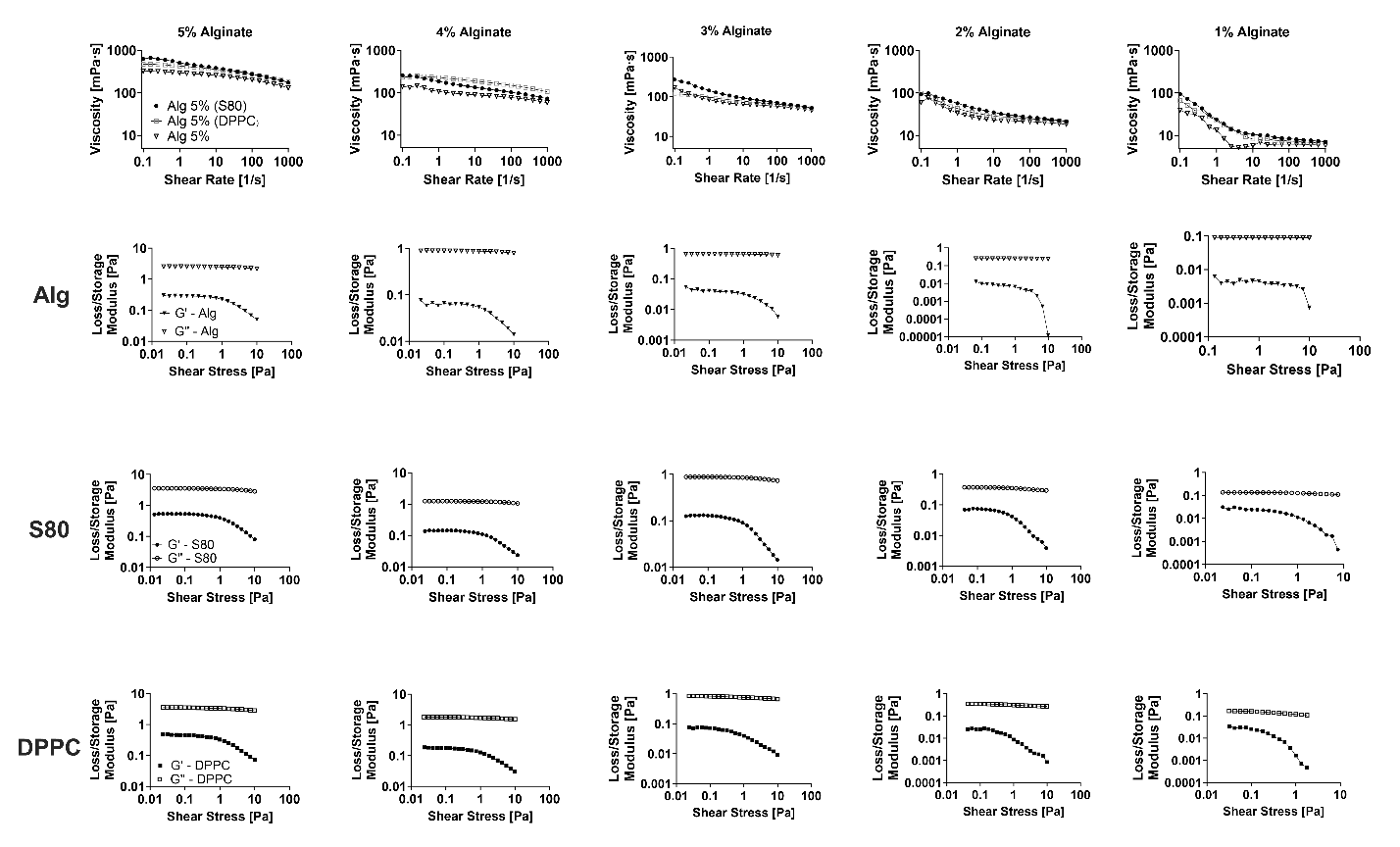


**Fig. S6.** Rheological properties of drug product inks. Viscosities curves and amplitude sweep experiments for all assessed formulations.
